# Interaction of Acetylcholinesterase with Neurexin-1β regulates Glutamatergic Synaptic stability in Hippocampal neurons

**DOI:** 10.1186/1756-6606-7-15

**Published:** 2014-03-05

**Authors:** Yun-Yan Xiang, Haiheng Dong, Burton B Yang, John F MacDonald, Wei-Yang Lu

**Affiliations:** 1 Robarts Research Institute, University of Western Ontario, London, Ontario, Canada; 2 Department of Physiology and Pharmacology, University of Western Ontario, London, Ontario, Canada; 3 Department of Anesthesia, University of Toronto, Toronto, Ontario, Canada; 4 Department of Laboratory Medicine and Pathobiology, University of Toronto, Toronto, Ontario, Canada; 5 Robarts Research Institute, The University of Western Ontario, 1151 Richmond Street North, London, Ontario N6A 5B7, Canada

**Keywords:** Protein interaction, Glycosylation, Neurodegeneration, Synaptic apoptosis

## Abstract

**Background:**

Excess expression of acetylcholinesterase (AChE) in the cortex and hippocampus causes a decrease in the number of glutamatergic synapses and alters the expression of neurexin and neuroligin, trans-synaptic proteins that control synaptic stability. The molecular sequence and three-dimensional structure of AChE are homologous to the corresponding aspects of the ectodomain of neuroligin. This study investigated whether excess AChE interacts physically with neurexin to destabilize glutamatergic synapses.

**Results:**

The results showed that AChE clusters colocalized with neurexin assemblies in the neurites of hippocampal neurons and that AChE co-immunoprecipitated with neurexin from the lysate of these neurons. Moreover, when expressed in human embryonic kidney 293 cells, *N*-glycosylated AChE co-immunoprecipitated with non-*O*–glycosylated neurexin-1β, with *N*-glycosylation of the AChE being required for this co-precipitation to occur. Increasing extracellular AChE decreased the association of neurexin with neuroligin and inhibited neuroligin-induced synaptogenesis. The number and activity of excitatory synapses in cultured hippocampal neurons were reduced by extracellular catalytically inactive AChE.

**Conclusions:**

Excessive glycosylated AChE could competitively disrupt a subset of the neurexin–neuroligin junctions consequently impairing the integrity of glutamatergic synapses. This might serve a molecular mechanism of excessive AChE induced neurodegeneration.

## Introduction

As the key enzyme that hydrolyzes acetylcholine, acetylcholinesterase (AChE) plays a critical role in regulating cholinergic signaling. Neurons in the central nervous system generate two isoforms of AChE: synaptic AChE (AChE-S, also known as “tailed AChE”) and read-through AChE (AChE-R). In adults, AChE-S is the predominant isoform, although AChE-R increases following exposure to a variety of stressors [1]. In the extracellular space, AChE exists in both soluble and membrane-bound forms. Soluble AChE includes monomeric AChE-R, as well as globular monomers and dimers of AChE-S. Membrane-bound AChE consists of AChE-S tetramers tethered to the cell membrane by a proline-rich membrane anchor [2]. Interestingly, membrane-bound AChE-S can be released in response to cholinergic activation [3]. Collective data imply that the isoforms, concentrations and localization of AChE within the brain are dynamically regulated.

In the brain, AChE is produced by cholinergic neurons [4], cholinoceptive neurons [5], and astrocytes [6]. Clinical studies have indicated that an increase in anomalous AChE is strongly correlated with the pathogenesis of Alzheimer disease (AD) [7-10]. Specifically, AChE is a major component of amyloid-β (Aβ) plaques [11], and *N-*glycosylated AChE is increased in the cerebrospinal fluid of patients with AD [12]. In particular, AChE binds to Aβ, thus promoting both the formation of Aβ fibrils [13] and the occurrence of neurotoxicity [14]. Moreover, augmenting AChE expression in the brains of transgenic mice that show neurodegeneration accelerates Aβ plaque formation [15]. However, the molecular mechanism or mechanisms by which anomalous AChE contributes to the pathogenesis of AD remain uncertain.

A progressive loss of synapses in the cortex and hippocampus is characteristic of early-stage AD [16]. Synaptic development and stability are regulated by the interaction of neurexin and neuroligin, transmembrane proteins that are expressed in the pre- and post-synaptic membrane domains of neurons, respectively [17,18]. These two proteins connect via their ectodomains to form a trans-synaptic junction. Neurexin and neuroligin also interact, via their cytoplasmic tails, with the PDZ domains of specific scaffolding proteins in the pre- and post-synaptic compartments, respectively. In this way, the neuroligin–neurexin junctions promote trans-synaptic adhesion and assist in the assembly of pre- and post-synaptic specializations [19]. Interestingly, the molecular sequence and three-dimensional structure of the ectodomain of neuroligins are homologous to the corresponding aspects of AChE [20]. We previously showed that over-expression of AChE alters the expression of neurexins and neuroligins and decreases the number of glutamatergic synapses in hippocampal neurons [21]. The objective of the present study was to determine if increased expression of AChE leads to a decrease in the neurexin–neuroligin junctions and consequently to a reduction in glutamatergic synapses.

## Results

### Interaction between AChE and neurexins in Hippocampal neurons

**Figure 1:**
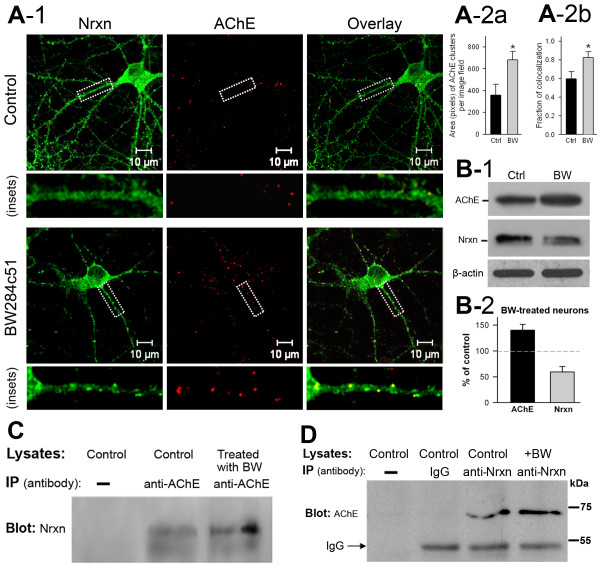
**Membrane-bound extracellular molecules of acetylcholinesterase (AChE) are clustered and co-localized with clustered neurexins in primary neurons. A-1**. Confocal microscopic images illustrating the intracellular localization of immunofluorescence of neurexins (Nrxn, left column) and membrane-bound extracellular AChE (middle column) in cultured hippocampal neurons under control conditions (top panel) and in neurons treated with the AChE inhibitor BW284c51 (lower panel). The right column illustrates the immunofluorescence overlay of the two proteins. Insets under each panel illustrate the subcellular distribution of the two proteins. Note co-localization of neurexin and AChE immunofluorescent clusters in neurites. **A-2a**. Total area (pixels) of AChE-immunoreactive clusters in control and BW284c51-treated neurons (control: 356 ± 100, n = 10 image fields; BW284c51: 682 ± 78, n = 10 image fields; *P* < 0.05). **A-2b**. Fraction of colocalization of AChE clusters with Nrxn clusters (control: 0.6 ± 0.08, n = 12 image fields; BW284c51: 0.82 ± 0.07, n = 12 image fields; *P* < 0.05). **B-1**. Immunoblots showing increase of AChE and decrease of neurexin in BW284c51-treated neurons. **B-2**. BW284c51treatment increased the total quantity of AChE (140% ± 11% of control) but decreased the total amount of neurexin (59% ± 10% of control). **C**. Co-immunoprecipitation assay of lysates of control neurons and neurons treated with BW284c51. The immunoprecipitating AChE co-precipitates with neurexin (Nrxn, middle lane), and BW284c51 enhances this co-precipitation (right lane). **D**. Assay for co-immunoprecipitating AChE using a neurexin antibody from lysates of control neurons under various control conditions. From left to right, lane 1: control neuron lysate without anti-neurexin antibody (anti-Nrxn); lane 2: control neuron lysate with IgG; lane 3: control neuron lysate with anti-neurexin; lane 4: BW284c51-treated neuron lysate with anti-neurexin. Note co-precipitation of AChE in lane 3 and increase in co-precipitation of AChE in BW284c51-treated neurons.

**Figure 2:**
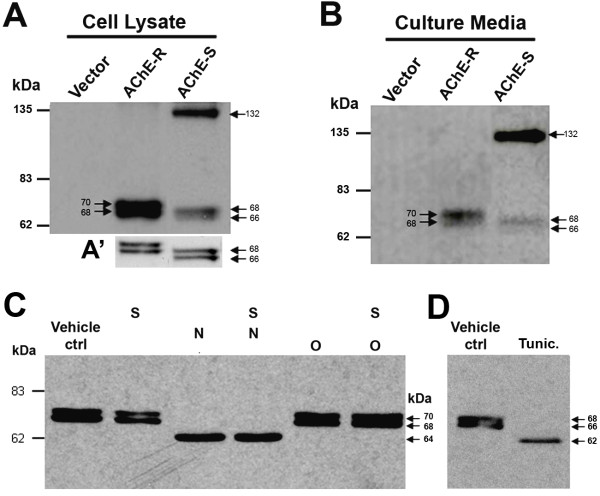
**Expression profile and glycosylation pattern of human acetylcholinesterase (hAChE) in human embryonic kidney 293 (HEK293) cells.** Expression profiles of read-through AChE (AChE-R) and synaptic AChE (AChE-S) in the cell lysate **(A)** and culture medium **(B)** of HEK293 cells transfected with hAChE-R or with hAChE-S. The AChE-S proteins display a band with molecular weight of approximately132 kDa, and another two bands with molecular weight of about 68 and 66 kDa, respectively, whereas the AChE-R proteins display two bands with molecular weight of about 70 and 68 kDa, respectively. **A’** illustrates the two lower molecular weight bands of AChE-S and AChE-R. **C**. Immunoblots of AChE-R generated by processing the cell lysates with various *N*- and *O*-glycohydrolases (N = peptide *N*-glycosidase F, an *N*-linked glycohydrolase; O = *O*-glycosidase and S = sialidase, two *O*-linked glycohydrolases). **D**. Immunoblots of AChE-S generated by processing lysates of transfected HEK293 cells without additional treatment (vehicle control [ctrl]) or with tunicamycin treatment (Tunic.).

We first investigated whether AChE interacts physically with neurexins. Specifically, we performed immunostaining for extracellular AChE in living neurons, followed by counterstaining for neurons under membrane-permeabilized conditions. Consistent with the results of an earlier study [22], we found that membrane-bound extracellular AChE molecules assembled in small bunches along neurites and were also found in larger patches associated with the perikaryon (Figure 1A-1, middle panels). In contrast, as previously reported [23], neurexin immunoreactivity was diffusely distributed in the perikarya, with small clusters in some neurites (Figure 1A-1, left panels). AChE immunoreactive particles were co-localized primarily with immunofluorescent clusters of neurexin (Figure 1A-1, right panels). Treating neurons with the AChE inhibitor BW284c51 decreased the fluorescent intensity of dispersed neurexins in most subcellular compartments but increased the fluorescent intensity of neurexin clusters in neurites (Figure 1A-1, left panel, inset). Inhibition of AChE activity increases the expression of AChE [24]. Indeed, treating the neurons with BW284c51 significantly increased the size of AChE particles (Figure 1A-1, middle panels, and Figure 1A-2a) and increased co-localization fraction of AChE with neurexin clusters (Figure 1A-1, right panels, and Figure 1A-2b). Immunoblotting assays (Figure 1B-1) confirmed that BW284c51 treatment increased AChE expression but decreased neurexin expression in neurons (Figure 1B-2).We then conducted reciprocal immunoprecipitation of neurexin and AChE from lysates of cultured hippocampal neurons. These assays demonstrated that immunoprecipitation of AChE using an AChE antibody led to co-precipitation of neurexin proteins which exhibited two bands; and BW284c51 treatment increased this co-precipitation (Figure 1C). In turn, a neurexin antibody, but not IgG protein, co-precipitated a single AChE band at about 68 kDa (Figure 1D). These results implied that AChE molecules are able to interact with some neurexin molecules in primary hippocampal neurons.

### Regulation of AChE–neurexin interaction by protein glycosylation

Mammalian neurons express both AChE-R and AChE-S, with each isoform displaying distinctive properties in molecular assemblies [25]. To study which AChE isoform interacts with neurexin-1β, we expressed Nrxn-1β-1’-*His* with either hAChE-S or hAChE-R in HEK293 cells. We began with outlining the expression profiles of these proteins in the transfected cells.

Consistent with the results of a previous study [26], immunoblotting the lysates of hAChE-S transfected cells, using anti-AChE, revealed a dense band at molecular weight about 136 kDa (Figure 2A, right lane), as well as two lighter bands at molecular weights about 66 and 68 kDa, respectively (see illustrations in Figure 2A’). The 66- and 68-kDa bands correspond to monomers of AChE-S [27-29], whereas the 136-kDa band may represent dimers of AChE-S. Blotting the lysates of hAChE-R transfected cells with anti-AChE also revealed two protein bands at molecular weights about 68 and 70 kDa (Figure 2A, middle lane; also see Figure 2A’), both of which should be globular monomers, as hAChE-R lacks the domain for polymerization. In addition, immunoblotting assays revealed that the hAChE-S and hAChE-R proteins had very similar profiles in the culture medium of transfected HEK293 cells (Figure 2B). Ellman esterase assays revealed that under our experimental conditions, the activity of hAChE in the culture media was about 1.0–1.5 units/ml for hAChE-S and 2.0 units/ml for hAChE-R.

To study the glycosylation pattern of AChE in mammalian cells, lysate of HEK293 cells transfected with AChE-R was treated with *N*- or *O*-glycohydrolases, both separately and in combination (Figure 2C). In another set of experiments, HEK293 cells transfected with AChE-S were treated with the *N*-glycosylase inhibitor tunicamycin (Figure 2D). Notably, immunoblotting of AChE-R from the cell lysate treated with the *N*-glycohydrolase PNGase F (Figure 2C) or from the lysate of AChE-S transfected cells treated with tunicamycin (Figure 2D) disclosed a single protein band, representing non-glycosylated AChE monomers. Specifically, the molecular weight of non-glycosylated AChE-R was about 64 kDa (Figure 2C), whereas the molecular weight of non-glycosylated AChE-S was about 62 kDa (Figure 2D). In contrast, immunoblotting AChE from the cell lysates treated with the *O*-linked glycohydrolases *O*-glycosidase and sialidase, separately or in combination, revealed two protein bands with the same molecular weights as the control (Figure 2C). These results confirm the previously reported finding that AChE molecules are highly modified by *N*-glycosylation [26,30,31], but not by *O*-glycosylation.

**Figure 3:**
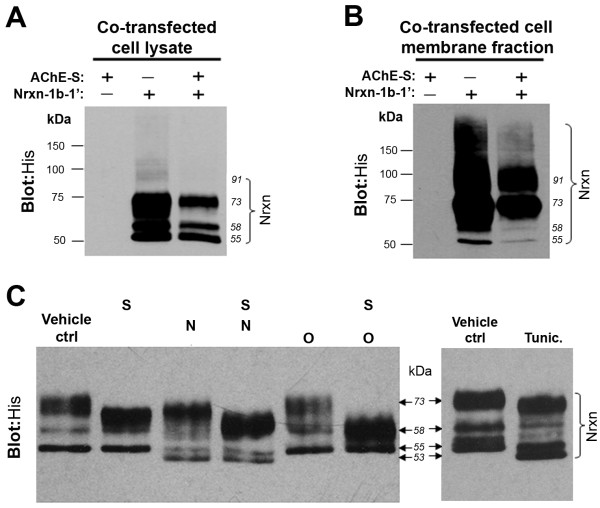
**Expression profile and glycosylation pattern of neurexin-1β in human embryonic kidney 293 (HEK293) cells. A**. Expression profiles of neurexin-1β-1’ (Nrxn -1β-1’) in total cell lysate of HEK293 cells that had been transfected with neurexin-1β-1’-*His* or synaptic acetylcholinesterase (AChE-S) or both. Neurexin-1β-1’ proteins were confirmed by anti-*His*. **B**. Expression profiles of neurexin-1β-1’ in the membrane fractions of HEK293 cells that had been transfected with neurexin-1β-1’-*His* alone or AChE-S alone or both. In both **A** and **B**, blotting *His* revealed multiple bands of neurexin-1β-1’. Notably, co-transfection with AChE decreased the expression of neurexin-1β in the HEK293 cells. **C**. Left panel: Immunoblots of neurexin-1β-1’ generated by processing the cell lysates with various *N*- and *O*-glycohydrolases (N = peptide *N*-glycosidase F, an *N*-linked glycohydrolase; O = *O*-glycosidase and S = sialidase, two *O*-linked glycohydrolases). Right panel: Immunoblots of neurexin-1β-1’ generated by processing lysates of transfected HEK293 cells without additional treatment (vehicle control [ctrl]) or with tunicamycin treatment (Tunic.). Note the similar change in molecular weight of the neurexin-1β-1’ bands following treatment with *N*-glycosidase F and tunicamycin.

By immunoblotting *His*, four major bands of neurexin-1β-1’, with molecular weights of about 55, 58, 73 and 91 kDa, respectively, were identified in total cell lysates (Figure 3A) and in the membrane fraction (Figure 3B) of HEK293 cells transfected with neurexin-1β-1’ alone or with AChE-S. Notably, in the cell lysate the 91-kDa band was faint whereas the 73-kDa band was dense (Figure 3A). In the membrane fraction, both the 91- and 73-kDa bands were strong, whereas the 55- and 58-kDa bands were less intense (Figure 3B). Interestingly, co-expression of AChE and neurexin-1β cDNA reduced the expression of neurexin-1β (Figures 3A and 3B, right lane) but did not affect the level of expression of AChE (not shown). These results are in accord with previous *in vivo* findings that over-expression of AChE decreases the expression of neurexin [32].

We also studied the glycosylation pattern of neurexin-1β in HEK293 cells. Our immunoblotting assays showed that in the total cell lysates treated with the *O*-glycohydrolases *O*-glycosidase and sialidase, the 73-kDa band of neurexin-1β disappeared while the density of the 58-kDa band of neurexin-1β increased dramatically (Figure 3C, left panel). These results indicate that the 73 kDa neurexin-1β band represents *O*-glycosylated forms. Notably, treating the transfected cells with tunicamycin (Figure 3C, right panel) and treating the lysates of transfected cells with PNGase F (Figure 3C, left panel) caused a slight reduction in the molecular weight of all neurexin-1β protein bands, from 55, 58 and 73 kDa, respectively, to about 53, 56 and 71 kDa, respectively. These results imply that short sugar chains are linked to *O*-glycosylated forms of neurexin-1β proteins by *N*-glycosylation, with the 53-kDa band representing the non-glycosylated form. Collectively, our results confirm a previous finding that neurexin-1β has minor *N*-glycosylation, and the various molecular weights of neurexin-1β primarily reflect various degrees of *O*-glycosylation [33]. In addition, our assays indicate that a large amount of neurexin-1β proteins located in the cell membrane is *O*-glycosylated.

**Figure 4:**
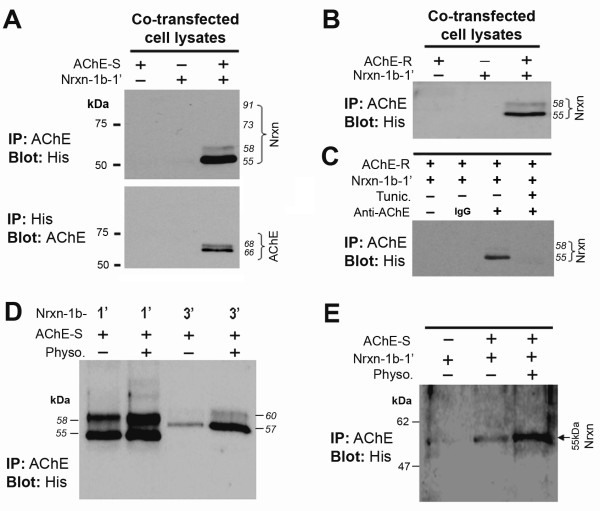
**Co-immunoprecipitation of neurexin-1β with acetylcholinesterase (AChE). A**. Representative blots of co-immunoprecipitation of neurexin-1β with AChE from lysate of HEK293 cells transfected with neurexin-1β-1’-*His* (Nrxn-1β-1’, lacking alternatively spliced sequence 4 [SS4]) alone or AChE-S alone or both. Upper panel: Immunoprecipitation of AChE-S from the lysate of HEK293 cells transfected with both neurexin-1β-1’-*His* and AChE-S co-precipitated a large amount of 55-kDa neurexin-1β-1’ and a small amount of 58-kDa neurexin-1β-1’ but did not co-precipitate the *O*-glycosylated 73- and 91-kDa forms. The co-precipitated neurexin-1β-1’ was confirmed by blotting *His.* Lower panel: Immunoprecipitation of *His* co-precipitated AChE-S from the lysate of cells transfected with both neurexin-1β-1’-*His* and AChE-S. **B**. Immunoprecipitation of AChE-R co-precipitated a large amount of 55-kDa neurexin-1β-1’ and a small amount of 58-kDa of neurexin-1β-1’ from the lysate of cells transfected with both neurexin-1β-1’-*His* and AChE-R. **C**. In control experiments, absence of AChE antibody (lane 1) and application of IgG (lane 2) led to absence of co-precipitation of neurexin-1β-1’. Similarly, when the transfected cells were cultured in the presence of tunicamycin (Tunic.), no neurexin-1β-1’ was co-precipitated by anti-AChE (lane 4). **D**. Co-precipitation of neurexin-1β by anti-AChE from lysate of cells transfected with AChE-S combined with neurexin-1β-1’ (from left to right: lanes 1 and 2) or combined with neurexin-1β-3’ (with SS4) (lanes 3 and 4). The transfected cells were cultured in the absence (lanes 1 and 3) or presence (lanes 2 and 4) of 10 μM physostigmine (Physo.). Neurexin-1β was confirmed by blotting *His*. The presence of physostigmine increased co-precipitation of neurexin-1β by anti-AChE, and the amount of co-precipitated neurexin-1β-3’ was less than the amount of neurexin-1β-1’. **E**. Immunoprecipitation of AChE from the lysates of co-cultured HEK293 cells transfected with either Nrxn-1β-1’ or AChE-S before co-culturing. Anti-AChE co-precipitated the 55-kDa neurexin-1β-1’ from co-cultured the cells, and physostigmine increased co-precipitation of neurexin-1β-1’.

We then examined whether neurexin-1β could be co-precipitated with AChE from lysates of HEK293 cells that had been co-transfected with Nrxn-1β-1’-*His* and either hAChE-S or hAChE-R. Immunoprecipitating either AChE-S (Figure 4A, lane 3 in upper panel) or AChE-R (Figure 4B, lane 3) led to co-precipitation of a large amount of 55-kDa Nrxn-1β-1’ and a small amount of 58-kDa Nrxn-1β-1’, but did not lead to co-precipitation of 73-kDa Nrxn-1β-1’ (Figures 4A and B). Conversely, immunoprecipitation of Nrxn-1β-1’ using anti-*His* antibody led to consistent co-precipitation of both 66- and 68-kDa monomers of hAChE-S (Figure 4A, lane 3 in lower panel). In the control experiment, neurexin-1β was not co-precipitated when the anti-AChE antibody was replaced with IgG (Figure 4C, lane 2). Remarkably, when the transfected cells were cultured in the presence of tunicamycin, immunoprecipitation of AChE did not lead to co-precipitation of neurexin-1β (Figure 4C, lane 4). Together, these results indicate that 1) both AChE-S and AChE-R can interact with a subset of neurexin-1β proteins that retain only *N*-linked short sugar chains and 2) *N*-glycosylation of AChE and neurexin-1β is required for interaction between these two molecules.

### Modulation of AChE–neurexin interaction by β-neurexin splicing and AChE ligand

Interaction of neurexins with neuroligins decreases when the 30 amino acid insert SS4 is present in the laminin G domain of β-neurexins [34]. To determine whether SS4 affects the interaction AChE with neurexin-1β, we co-immunoprecipitated the lysates of two sets of HEK293 cells: one set of cells transfected with hAChE-S and Nrxn-1β-1’-*His* (without SS4) and another set of cells transfected with hAChE-S and Nrxn-1β-3’-*His* (with SS4) using anti-AChE. Similar to the non-*O*-glycosylated Nrxn-1β-1’ (Figure 4D, lanes 1 and 2)*,* non-*O*-glycosylated Nrxn-1β-3’ molecules were also co-precipitated by anti-AChE, displaying as two bands at molecular weights of about 57 and 60 kDa, respectively (Figure 4D, lanes 3 and 4). However, at the same quantity of cell lysate proteins, the amount of co-precipitated Nrxn-1β-3’ (Figure 4D, lane 3) was less than that of co-precipitated Nrxn-1β-1’ (Figure 4D, lane 1). This result implies that alternative splicing of the SS4 in Nrxn-1β regulates the interaction between AChE–neurexin to some extent.

Surface anionic residues expressed on the esterase side of AChE allow this molecule to interact with various proteins [35-38]. To study whether some AChE ligands and/or inhibitors regulate interactions between AChE and neurexin, we examined co-immunoprecipitates of lysates of A549 cells transfected with hAChE-S combined with either Nrxn-1β-1’-*His* or with Nrxn-1β-1’-*His* in the absence or presence of the AChE inhibitor physostigmine (10 μM, added to the culture medium). Interestingly, physostigmine enhanced co-precipitation of AChE-S with neurexin-1β-1’ and with neurexin-1β-3’ (Figure 4D, lanes 2 and 4), which suggests that the AChE ligand may structurally regulate the interaction of AChE with neurexin.

### AChE interacts only with neurexin-1β located in cell membrane

To test our hypothesis that AChE interacts with the ectodomain of β-neurexin, we transfected one set of HEK293 cells with Nrxn-1β-1’-*His* and another set of HEK293 cells with AChE-S. Sixteen hours after transfection, we co-cultured the two sets of transfected cells for another 24–32 hours in the absence or presence of physostigmine. Immunoprecipitating the lysate of these co-cultured cells with anti-AChE led to co-precipitation of the 55-kDa Nrxn-1β-1’ protein, which was confirmed by blotting *His* (Figure 4E, lane 2). Again, physostigmine increased the band density of co-precipitated Nrxn-1β-1’ (Figure 4E, lane 3). This result confirms that AChE interacts with the ectodomain of neurexin-1β. In another experiment, we mixed the lysate of cells transfected with AChE-S with the lysate of cells transfected with Nrxn-1β-1’-*His*. In this test, anti-AChE did not co-precipitate any Nrxn-1β-1’ from the cell lysate mixture, even in the presence of physostigmine (not shown). Together, these combined results suggest that AChE interacts only with β-neurexins located on the cell membrane.

### Excess AChE decreases the neurexin–neuroligin association

**Figure 5:**
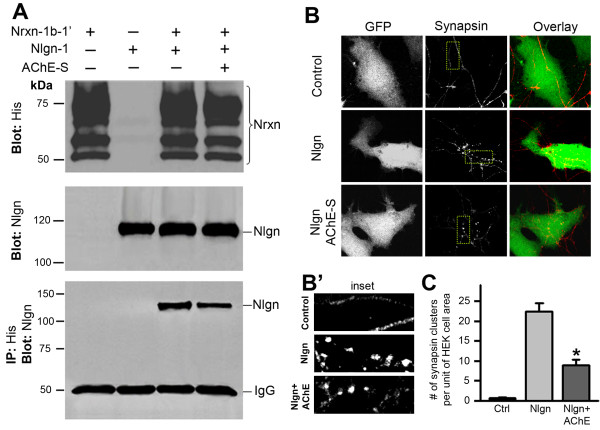
**Excess extracellular AChE decreases the association between neurexin and neuroligin and inhibits neuroligin-induced presynaptic specialization. A**. Cell lysates were prepared from HEK293 cells transfected with neurexin-1β-1’-*His* (Nrxn-1β-1’) alone (lane 1) or neurogligin-1 (Nlgn-1) alone (lane 2) or both (lanes 3 and 4). Neurexin-1β-1’ was proved by blotting *His* (top panel), and neuroligin-1 was confirmed by a neuroligin antibody (middle panel). Immunoprecipitation of *His* co-precipitated a heavy band of neuroligin from the lysate of cells expressing both proteins (upper band in lane 3 of bottom panel). Immunoprecipitating *His* co-precipitated a smaller band of neuroligin from the lysate of cells cultured in AChE-conditioned medium (upper band in lane 4 of bottom panel). **B**. Images of synapsin in primary neurons co-cultured with HEK293 cells transfected with or without neuroligin-1. Left column, first row: HEK293 cells transfected with GFP alone; second row: Cells transfected with GFP and neuroligin-1; third row: Cells transfected with GFP and neuroligin-1 and treated with AChE-conditioned medium. Middle column: Axonal neurites of hippocampal neurons were identified by immunostaining of synapsin. No synapsin clusters were seen in axonal neurits passing through GFP-expressing cells (Control; top row); whereas dense synapsin-clusters were formed on neuroligin-transfected cells (Nlgn; middle row). Less synapsin-clusters were formed on neuroligin-transfected cells grown in AChE-conditioned medium (Nlgn + AChE; bottom row). Right column; Overlay images of GFP-expressing cells and synapsin-positive structures. **B’**. Insets illustrate synapsin-clusters associated with control cells (top), Nlgn-expressing cells (mid) and Nlgn-expressing cells in AChE-conditioned medium (bottom). **C**. Number of synapsin-positive clusters in single cells transfected with vector (Ctrl, 0.8 ± 0.2, n = 36 cells), with neuroligin-1 (Nlgn, 22.5 ± 2, n = 19) or with neuroligin-1 and in AChE-conditioned medium (Nlgn + AChE, 8.7 ± 1.3, n = 22, * *P* < 0.05, relative to conditions without AChE).

Excess AChE proteins binding to β-neurexins may reduce the association of neurexin with neuroligin. To test this possibility, we performed co-immunoprecipitation assays of the lysate of HEK293 cells expressing Nrxn-1β-1’-*His* and neuroligin-1. The cells were then cultured in medium without (control) or with added AChE-S (1 unit/ml). As expected, neuroligin-1 was co-immunoprecipitated with Nrxn-1β-1’ (Figure 5A, lane 3, bottom panel). Culturing the transfected cells in the presence of AChE-S significantly reduced the amount of co-immunoprecipitated neuroligin (Figure 5A, lane 4, bottom panel). This result indicates that excess extracellular AChE indeed disrupts the association of β-neurexins and neuroligin-1.

Next, we tested whether the disruption of the neurexin–neuroligin association caused by excess AChE leads to interruption of synaptic stability. Axonal terminals that pass through non-neuronal cells transfected with neuroligin-1 initiate presynaptic differentiation, expressing presynaptic proteins and forming presynaptic structures; such synaptogenic activities are initiated by the interaction between β-neurexin and neuroligin [39]. Using this cellular model, we examined whether excess AChE in the extracellular space could alter the neurexin-neuroligin interaction induced presynaptic maturation. As previously reported [39], we found that the axonal neurites of embryonic hippocampal neurons developed synapsin-immunoreactive presynaptic buttons on HEK293 cells expressing neuroligin-1 (Figure 5B, middle row), and such button-like presynaptic structures were not formed by axonal neurites on control HEK293 cells (Figure 5B, top row). This result confirms that neuroligin-1 does indeed initiate development of presynaptic structures. Importantly, we found that culturing the neuroligin-1-expressing HEK293 cells in the AChE-S-conditioned medium (with 5 μM physostigmine) greatly decreased the number of synapsin-immunoreactive presynaptic buttons on neuroligin-expressing cells (Figures 5B and C). Together, these results imply that excess extracellular AChE could inhibit synaptic development or interrupt synaptic stability, by reducing the association of β-neurexin with neuroligin.

### Excess AChE reduces glutamate-induced currents in Hippocampal neurons

**Figure 6:**
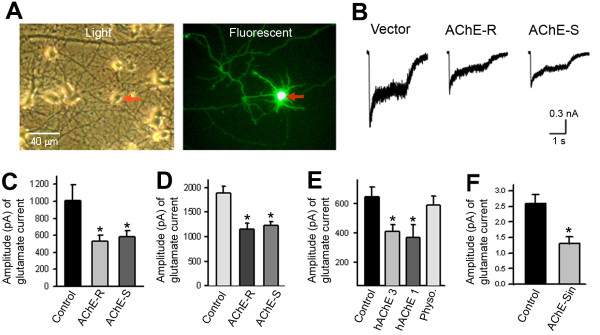
**Increase in extracellular acetylcholinesterase (AChE) decreases glutamate-induced currents in primary neurons. A**. Representative images of cultured hippocampal neurons transfected with complementary DNA encoding AChE. Left panel shows cultured neurons under a light microscope, and right panel displays a GFP fluorescent image of the same field. Red arrow indicates the cell body of a transfected cell. **B**. Traces of glutamate-induced current in a control (Vector) neuron, a neuron expressing read-through AChE (AChE-R) and a neuron expressing synaptic AChE (AChE-S). **C**. Amplitude of glutamate-induced current in control neurons (expressing GFP only), AChE-R-expressing and AChE-S-expressing neurons (control: 1013 ± 181 pA, n = 11 neurons; AChE-R: 532 ± 69 pA, n = 11 neurons; AChE-S: 580 ± 72 pA, n = 12 neurons; * *P* < 0.05). **D**. Amplitude of glutamate-induced current in control neurons and in neurons surrounding the AChE-R- and AChE-S-expressing neurons (control: 1881 ± 146 pA, n = 7 neurons; surrounding AChE-R-expressing neurons: 1157 ± 116 pA, n = 7 neurons; surrounding AChE-S-expressing neurons: 1236 ± 69 pA, n = 7 neurons; * *P* < 0.05). **E**. Amplitude of glutamate-induced current in control neurons and in neurons treated with 3.0 units/ml human AChE-S (hAChE 3), 1.0 unit/ml human AChE-S (hAChE 1) or 10 μM physostigmine (Physo) (control: 645 ± 68 pA, n = 11 neurons; hAChE 3: 413 ± 45 pA, n = 11 neurons; hAChE 1: 367 ± 90 pA, n = 9 neurons; Physo.: 587 ± 62 pA, n = 10 neurons; * *P* < 0.05). **F**. Amplitude of glutamate-induced current in control neurons and in neurons treated with conditioned medium containing catalytically inactive AChE-S (AChE-S*in*) (control: 2.6 ± 0.29 nA, n = 8 neurons; AChE-Sin: 1.3 ± 0.22 nA, n = 11 neurons; * *P* < 0.05).

To examine whether increased expression of AChE impairs synaptic function in neurons, we expressed hAChE-S or hAChE-R in hippocampal neurons that had been cultured in the medium containing 10 μM physostigmine. Neurons expressing hAChE and GFP together or GFP alone (control) were identified by green fluorescence (Figure 6A), and these GFP-expressing neurons accounted for about 0.5%–1.5% of the total neuron population. Patch-clamp recordings showed that the amplitude of glutamate-induced currents in neurons expressing hAChE-S or hAChE-R was significantly lower than the amplitude of these currents in control neurons (Figures 6B and C). In contrast, the amplitude of GABA-evoked currents in AChE-expressing neurons was comparable to that of GABA-evoked currents in control neurons (not shown). We also measured glutamate currents in non-transfected neurons closely surrounding the transfected neurons (with cell-body-to-cell-body distance of 50–200 μm). Interestingly, glutamate currents in these non-transfected neurons were also lower than those of control neurons (Figure 6D). These results indicate that increased expression of AChE reduces glutamate receptor activity, likely through one or more extracellular mechanisms. Indeed, adding 1.0–3.0 units/ml purified hAChE-S and 10 μM physostigmine to the culture medium also decreased the glutamate currents (Figure 6E). Furthermore, treating the neurons with conditioned medium containing catalytically inactive AChE-S (AChE-Sin) also substantially decreased glutamate currents (Figure 6F). These results indicate that excess extracellular AChE jeopardizes the glutamatergic function of neurons through an extracellular mechanism that is independent of its catalytic function.

### Excess extracellular AChE decreases glutamatergic synapses in primary neurons

**Figure 7:**
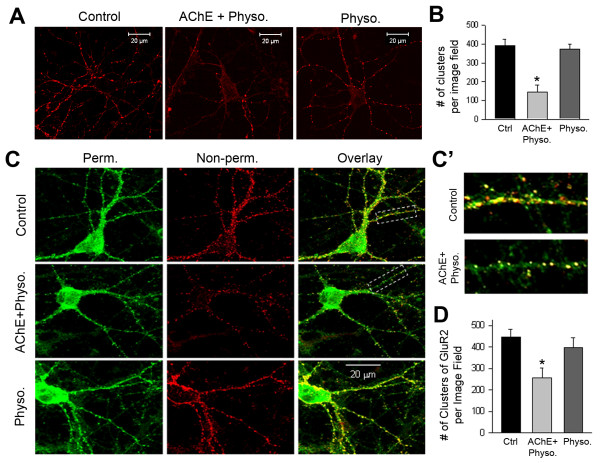
**Excess extracellular acetylcholinesterase (AChE) decreases glutamatergic synapses. A**. Confocal microscopic images showing immunofluorescent staining of synapsin in cultured hippocampal neurons at the 12th day *in vitro* (DIV). Panels show, from left to right, neurons in control medium, in synaptic AChE (AChE-S)-conditioned medium containing 10 μM physostigmine (AChE + physo.) and in control medium containing physostigmine (physo. 10 μM). White bar represents 20 μm. **B**. Numbers of synapsin-positive clusters per image field in neurons grown under various conditions (control: 392 ± 26 clusters/field, n = 12 images; AChE + physo: 141 ± 33 clusters/field, n = 15 images; Physo. 381 ± 22 clusters/field, n = 9 images; * *P* < 0.05). **C**. Immunofluorescent staining of the GluR2 subunit of the glutamate receptor in cultured hippocampal neurons at the 12th DIV. Rows represent neurons gown under various culture conditions: top row: control medium; middle row: AChE-conditioned medium + 10 μM physostigmine; bottom row: physostigmine alone. The GluR2 subunits on the membrane surface were stained first under non-permeabilizing conditions (red, middle column) and the intracellular GluR2 subunits were then stained under permeabilizing conditions (green, left column). Right column shows overlay images of surface and intracellular GluR2 subunits. **C’**. Enlarged images of the areas enclosed by a dotted line in C, illustrating GluR2 clusters in control and AChE-treated neurons. Notably, increased extracellular AChE reduced the number of surface GluR2 subunit clusters but did not affect the immunoreactivity of intracellular GluR2 subunits. **D**. Numbers of surface GluR2-positive clusters per image field in neurons cultured in various media (control: 440 ± 28 clusters/field, n = 8 images; AChE + physo.: 256 ± 50 clusters/field, n = 12 images; Physo.: 399 ± 48 clusters/field, n = 8 images; * *P* < 0.05).

**Figure 8:**
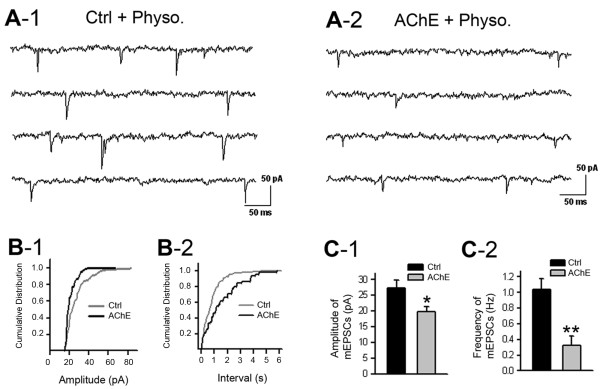
**Excess extracellular acetylcholinesterase (AChE) decreases glutamatergic synaptic activities. A**. Representative traces of miniature excitatory postsynaptic currents (mEPSCs) recorded at the 11th day *in vitro* in neurons grown in control medium **(A-1)** and in synaptic AChE (AChE-S)-conditioned medium with 10 μM physostigmine **(A-2)**. **B**. Cumulative distribution of the amplitude **(B-1)** and interval **(B-2)** of mEPSCs recorded in a neuron cultured in control medium (Ctrl) and in a neuron grown in AChE-S-conditioned medium with physostigmine (AChE). **C**. Amplitude **(C-1)** and frequency **(C-2)** of mEPSCs recorded in a group of neurons cultured in control medium (Ctrl) and in neurons grown in AChE-S-conditioned medium with physostigmine (AChE). Amplitude under control conditions: 25.5 ± 2.5 pA, n = 6 cells; amplitude under high-AChE conditions: 20 ± 2 pA, n = 8 cells; frequency under control conditions: 1.04 ± 0.15 Hz, n = 6 cells; frequency under high-AChE conditions: 0.32 ± 0.14 Hz, n = 8 cells. * *P* < 0.05, ** *P* < 0.001.

We then examined whether excess extracellular AChE impairs glutamatergic synapses in primary neurons. Specifically, we treated cultured hippocampal neurons at the ninth DIV with AChE-S-conditioned medium and 10 μM physostigmine. At the 12th DIV, we performed immunostaining for synapsin and the GluR2 (GluA2) subunit of glutamate receptors. These assays showed that the increase in AChE-S was associated with significant reductions in the number of synapsin clusters (Figures 7A and B) and the expression of GluR2 (Figure 7C and 7D) along dendritic neurites, indicating a reduction in the number of glutamatergic synapses. To investigate the effect of increased AChE on synaptic activity, we recorded mEPSCs in cultured hippocampal neurons grown in control medium (Figure 8A-1) or in AChE-S-conditioned medium (Figure 8A-2). Increasing AChE in the culture medium significantly reduced the frequency and amplitude of the mEPSCs (Figures 8A-C).

## Discussion

The activity and expression levels of AChE control the amount of extracellular acetylcholine, which critically regulates glutamatergic synaptic transmission. AChE has non-catalytic functions *in vitro*[40,41] and *in vivo*[42], some of which likely involve protein–protein interactions [1]. Consistent with this notion, the present study revealed that excess *N*-glycosylated AChE alters the structure and function of glutamatergic synapses through its interaction with non-*O*-glycosylated neurexins and a consequent reduction in the association between neurexin and neuroligin. These findings delineate a molecular mechanism whereby excess glycosylated AChE could contribute to neurological disorders in the brain. Previous studies have shown that increased expression of AChE in cortical neurons reduces the number of synapses and the levels of β-neurexins both *in vivo*[43,44] and *in vitro*[21]. These results suggest crosstalk between AChE and neurexin. We hypothesized that these two proteins might interact physically and anticipated that they would be co-located in primary neurons. Indeed, our immunocytochemical assays showed that many AChE assemblies in neurites were co-localized with neurexin clusters (Figure 1A-1), which implies a potential *in situ* association between the two molecules. In addition, AChE could be reciprocally co-precipitated with neurexin-1β from the lysates of cultured neurons (Figure 1B, middle lane). Furthermore, as previously reported [45], treating neurons with the selective AChE inhibitor BW284c51 enhanced the expression of AChE (Figure 1B) and increased the co-precipitation of AChE with neurexin (Figure 1C, right lane). Together, these results imply that interactions of AChE with neurexin occur in primary neurons.

The interaction between AChE and neurexin was further substantiated by assaying lysates of HEK293 cells that had been transfected with hAChE-R or AChE-S, either separately or in combination with Nrxn-1β-1’ or with Nrxn-1β-3’. Under natural conditions, AChE-R formed only monomers, whereas AChE-S existed as monomers and possibly dimers (Figures 2A and 2B). As previously reported [26], under denaturing conditions (i.e., in the presence of *N*-glycohydrolase), both isoforms of AChE appeared as glycosylated monomers with reduced molecular weight (Figure 2C). Under denaturing conditions, neurexin-1β in cell lysates and membrane fractions also exhibited glycosylated forms (Figures 3A and B). Specifically, a large amount of neurexin-1β proteins in HEK293 cells had large molecular weights (73 kDa) as they were associated with both *N*- and *O*-linked glycans, while a lesser quantity of neurexin-1β exhibited smaller molecular weights (55 and 58 kDa) because they had only *N*-glycosylation.

Remarkably, monomers of both AChE-S and AChE-R were reciprocally co-immunoprecipitated only with the *N*-glycosylated (55 and 58 kDa) neurexin-1β proteins, not with the *O*-glycosylated (73 kDa) forms (Figures 4A and B). These results are in accord with those of an earlier study in which no interaction of AChE with the 72-kDa neurexin-1β was detected [46]. However, our observation of co-immunoprecipitation of neuronal AChE variants with non-*O*-glycosylated β-neurexins was novel. Previous studies showed that AChE-S and AChE-R differ in structure of C-terminals and have inverse effects on amyloid fibrils formation [47,48], implying that the two neuronal AChE variants may have distinctive roles in AD pathology. Therefore, the significance of interactions between the non-*O*-glycosylated β-neurexins and the two AChE variants awaits further studies.

The AChE–β-neurexin interaction requires *N-*glycosylation of either AChE or neurexin-1β, because there was no co-precipitation of neurexin-1β with AChE when the *N*-glycosylases were inhibited (Figure 4C, lane 4). In addition, co-precipitation of AChE and neurexin was observed in the lysate of co-cultured cells that had been transfected with either AChE-S or Nrxn-1β-1’ before co-culturing (Figure 4E); in contrast, co-precipitation was not observed in a mixture of lysates from cells that were separately transfected with either AChE-S or Nrxn-1β-1’ (not shown). These results indicate that AChE monomers interact with the extracellular domain of β-neurexins, specifically those located in the cell membrane.

**Figure 9:**
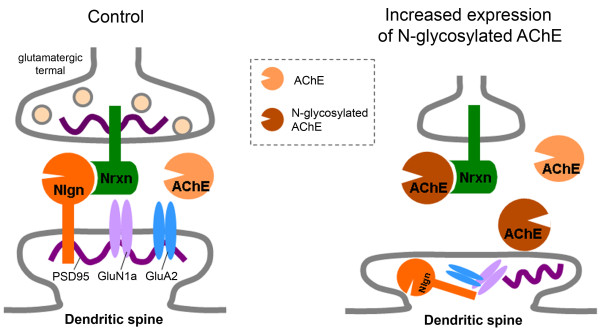
**Possible interactions among neurexin (Nrxn), neuroligin (Nlgn) and acetylcholinesterase (AChE) at glutamatergic synapses under normal and pathological conditions.** Under normal conditions, *O*-glycosylated β-neurexins are primarily associated with neuroligin-1, forming trans-synaptic junctions and inducing pre- and post-synaptic maturation. Under pathological conditions, *N*-glycosylated AChE increases (or *O*-glycosylation of β-neurexins decreases), allowing more interactions between *N*-glycosylated AChE and non-*O*-glycosylated β-neurexins and a consequent loss of glutamatergic synapses and intracellular accumulation of post-synaptic proteins such as postsynaptic density protein 95 (PSD-95) and the glutamate receptor subunits GluN1a (NR1a) and GluA2 (GluR2).

The enzymatic residues of AChE locate at the bottom of a “gorge” of the molecule [49]. The surface anionic residues surrounding the gorge are critical for AChE to interact with various proteins [35,36,50]. However, it is unlikely that the interaction between AChE and β-neurexins occurs via these surface anionic residues because BW284c51, a selective AChE inhibitor that specifically binds to the anionic residues [51] enhanced, rather than interrupted, co-immunoprecipitation of AChE with neurexin-1β (Figure 1C). Like AChE, the ectodomain of neuroligin-1 also possesses surface anionic residues. Given that binding of the ectodomain of neuroligin-1 to neurexin-1β occurs on the side opposite to the surface anionic residues [52], we propose that AChE similarly interacts with β-neurexins through a region on the opposite side of the surface anionic residues (see Figure 9). Physostigmine, an AChE inhibitor that binds to enzymatic residues in the gorge of AChE, also increased the interaction between neurexin-1β and AChE-S (Figures 4D and E). This effect of physostigmine is not likely to be due to its catalysis-inhibiting activity, because no innate cholinergic signaling mechanism is known to exist in HEK293 cells. Future structural studies will help to determine the molecular mechanisms by which these AChE ligands regulate the interaction between AChE and neurexin. The activity of AChE in the brain is critically associated with neural development and cognition [53]. AChE expression peaks in the fetus during the neuritogenic period before synaptogenesis [54] and gradually declines upon postnatal maturation [55,56]. However, in the adult brain, the expression of AChE increases in response to psychological stressors [57-59]. This increased AChE expression is detrimental to the synapses [43] and ultimately to brain function [60]. In spite of this, knowledge of the molecular mechanism by which excess AChE impairs the integrity of synapses has been lacking. The observation that AChE interacts with non-*O-*glycosylated β-neurexin led us to test the hypothesis that excess AChE might decrease the neurexin–neuroligin association at excitatory synapses. Indeed, adding AChE-S to the culture medium reduced the neurexin–neuroligin association (Figure 5A). Notably, the reduction was small, which may reflect the facts that AChE interacts only with a small amount of non-*O-*glycosylated β-neurexin proteins (see Figure 4A). Nevertheless, increasing extracellular AChE did largely inhibit the neuroligin-induced *de novo* formation of presynaptic structure (Figure 5B, bottom row). Moreover, including both AChE and AChE inhibitor in the culture medium significantly decreased glutamate-evoked currents (Figure 6), reduced the number of glutamatergic synapses (Figure 7) and lowered the frequency and amplitude of mEPSCs (Figure 8). Together, these results indicate that excess AChE could impair the integrity of some native glutamatergic synapses.

Although the association between neurexin and neuroligin is essential for both excitatory and inhibitory synapses [61], neuroligin varies in terms of the isoforms present at excitatory and inhibitory synapses. Specifically, neuroligin-1 is primarily localized at glutamatergic synapses [18], whereas neuroligin-2 is largely restricted to GABAergic synapses [62]. In the present study, AChE exhibited greater affinity for neurexin-1β-1’ (lacking the SS4 insert) (Figure 4D). Given that neurexin-1β-1’ has a higher affinity for neuroligin-1 and a lower affinity for neuroligin-2 [34], it is plausible that AChE preferentially disrupts the interaction between neurexin-1β-1’ and neuroligin-1 at glutamatergic synapses. Indeed, increased expression of AChE reduces the cell surface clusters of glutamate receptor subunits but increases the total proteins of neuroligin, glutamate receptor subunits and postsynaptic density protein 95 (PSD-95) in hippocampal neurons [21], which together imply that excess AChE decreases glutamatergic synapses and thus allows cellular accumulation of postsynaptic proteins. Nevertheless, the issue as to whether AChE does not disrupt the association between neurexin-1β and neuroligin-2 but selectively interrupts the association between neurexin-1β and neuroligin-1, requires more detailed studies.

Bi-directional signaling by adhesion molecules in the pre- and post-synaptic compartments determines the appropriate differentiation of synaptic structures [63]. The interaction of neurexins and neuroligins mediates signaling across the synapse and ultimately shapes the properties of neural networks [64]. Previous studies have demonstrated that glycosylation of neurexin-1β is required for such interactions [62]. In the current study, the majority of membrane-targeted β-neurexins were modified by both *O*- and *N*-glycosylation, whereas a small number of β-neurexins in the membrane were linked only with *N*-glycans (Figure 3). Interestingly, AChE monomers interacted only with the non-*O*-glycosylated β-neurexins (Figures 3 and 4), and such interactions might fine-tune synaptic stability (Figures 7 and 8), probably possibly through modulation of the neurexin–neuroligin association (Figure 5).

Another important outcome of this study was the finding that the AChE-neurexin interaction requires AChE *N*-glycosylation (Figure 4C, lane 4). Given that *N-*glycosylated AChE is significantly increased in the cerebrospinal fluid of patients with AD [12], and that the expression of AChE is negatively regulated by miR-132 [65], which largely decreases in late-onset AD patients [66], it is plausible that the interaction between AChE and neurexin increases in the brains of AD patients, leading to damage at the glutamatergic synapses. We propose that under normal physiological conditions, a low concentration of *N*-glycosylated AChE is present at glutamatergic synapses, which has little effect on the synaptic stability (Figure 9). However, under pathological conditions such as AD, the quantity of *N*-glycosylated AChE monomers in the brain increases greatly, to the point where the monomers interact with β-neurexins, adversely affecting the structure and function of a subset of glutamatergic synapses. Such action of excessive AChE may also link to other cognitive diseases, for example autism, because alterations in cholinergic activity [67] and/or in neuroligin-neurexin association [64] in the brain lead to autism spectrum disorders.

## Conclusion

In summary, our studies demonstrated that excess glycosylated AChE could interact with non-*O*-glycosylated neurexin-1β, thus competitively disrupting a subset of the neurexin–neuroligin junctions and consequently impairing the integrity of some glutamatergic synapses. Our findings may provide a molecular mechanism of excessive AChE induced neurodegeneration.

## Methods

### Complementary DNA construction

Complementary DNA (cDNA) encoding full-length human AChE-S (hAChE-S), inactive hAChE-S (hAChE-S_in_) and human AChE-R (hAChE-R) [68] cloned into the pL5CA expression vector were gifts from Dr. Hermona Soreq at the Hebrew University of Jerusalem. cDNA encoding a full-length rat neuroligin-1 [69] cloned in the pCMV5 expression vector (pCMVNL1), cDNA encoding a rat neurexin-1β lacking alternatively spliced sequence 4 (SS4) (neurexin-1β-1’) from pCMVN1β-1 and cDNA encoding a full-length rat neurexin-1β containing SS4 (neurexin-1β-3’) from pCMVN1β-3 [70] were gifts from Dr. Thomas C. Südhof at Stanford University. To obtain better immunobiochemical signals for neurexins, the neurexin cDNA samples were inserted into a pcDNA3 expression vector tagged with 6-*His*[71,72]. Briefly, the cDNA for full-length neurexin-1β-1’ and neurexin-1β-3’ was amplified by polymerase chain reaction using platinum *Pfx* DNA polymerase (Invitrogen, Carlsbad, CA) from pCMVN1ß-1 and pCMVN1ß-3 cDNA, respectively. The product of the polymerase chain reaction was inserted into the *BamHI-XbaI* site of the pcDNA3, positioned in frame upstream to the 6-*His* epitope and followed by a stop codon. All constructs were re-sequenced, with confirmation of the absence of nucleotide errors and the in-frame context of the neurexin cDNA with 6-*His*. cDNA encoding *His*-tagged rat neurexin-1β from those vectors was named neurexin-1β-1’-*His* (Nrxn-1β-1’-*His*) and neurexin-1β-3’-*His* (Nrxn-1β-3’-*His*), respectively.

### AChE-conditioned media

Human embryonic kidney 293 (HEK293) cells were maintained in Dulbecco’s modified Eagle medium (DMEM) supplemented with 10% fetal calf serum in a 37°C humidified atmosphere containing 5% CO_2_. Cells at 75% confluence were transiently transfected with cDNA encoding hAChE-S, hAChE-Sin or hAChE-R or with the expression vector alone, using Lipofectamine 2000 transfection reagent (Invitrogen), according to the manufacturer’s instructions. Twelve hours after transfection, the DMEM was replaced with fresh B27-supplemented Neurobasal medium (1:50, Invitrogen). Twenty-four hours later, the hAChE-conditioned media and the hAChE-free conditioned medium (from cells transfected with expression vector alone) were collected. Previous work has shown that recombinant hAChE is secreted from transiently transfected HEK293 cells as a soluble globular enzyme [73]. The presence of active hAChE in the conditioned medium was confirmed by an AChE activity assay according to Ellman’s method, as previously described [21]. The presence of catalytically inactive hAChE-S (hAChE-S_in_) in its conditioned medium was confirmed by immunoblot assays.

### Culture of Hippocampal neurons

Neuronal cell dissociation and culture were performed as previously described [21,74]. Briefly, hippocampal cells from Wistar rat embryos at day 18 (E18) were isolated by mechanical trituration. The dissociated cells were cultured on dishes or coverslips coated with poly-d-lysine (Sigma, Oakville, Canada) at a density of 3 × 10 [4] cells/cm^2^ in standard plating medium containing B27-supplemented Neurobasal medium, 0.5 mM l-glutamine, 25 μM glutamic acid, 0.5 mM sodium pyruvate and 0.5% fetal bovine serum. The hippocampal cells were incubated for 18 hours at 37°C in an atmosphere containing 5% CO_2_, after which the plating medium was replaced with culture medium containing B27-supplemented Neurobasal medium and l-glutamine (0.5 mM). This culture medium supports neuronal growth while restricting the growth of other cell types [75]. According to the requirements of specific experiments, the neurons were treated and used at various days *in vitro* (DIV).

To investigate whether an increase in AChE expression affects neuronal function, two sets of cultured hippocampal neurons at the fifth DIV were transfected (using Lipofectamine 2000 transfection agent) with cDNA, one set with cDNA encoding hAChE-S and the other set with cDNA encoding hAChE-R, along with a green fluorescent protein (GFP)-encoding vector. Neurons transfected with both the pL5CA expression vector and the GFP-encoding vector were used as controls. Three hours after transfection, the transfection medium was replaced with fresh B27-supplemented Neurobasal medium. To eliminate the catalytic action of excessive AChE, the AChE inhibitors physostigmine (10 μM, Sigma) or 1, 5-bis (4-allyldimethylammoniumphenyl)-pentan-3-one dibromide (BW284c51, 5 μM, Sigma) was added to the media. Twelve to fourteen hours after transfection, a fluorescence microscope was used to identify the transfected neurons (labeled with GFP fluorescence), and the transfection rate was calculated. To study whether excess AChE impairs neuronal function by an extracellular mechanism, other sets of cultured hippocampal neurons at the fifth DIV were treated with AChE-conditioned media or medium to which purified hAChE-S had been added (Sigma, C-1682; generated from the same hAChE cDNA used in this study) in the presence of physostigmine (10 μM).

### Immunocytochemical assays in cultured neurons

Immunocytochemical assays were performed as previously described [21,74], with minor modifications. Briefly, live cultured hippocampal neurons at the 11th DIV, with or without BW284c51 treatment (5 μM, for 3 days), were incubated with anti-AChE antibody (Chemicon, Temecula, CA, diluted 1:100 in the medium) for 2 hours. After washing with phosphate-buffered saline (PBS), the neuronal cells were fixed with 3.7% paraformaldehyde and 4% sucrose in PBS for 10 minutes. Following incubation with a Cy3-conjugated donkey anti-mouse secondary antibody (Jackson ImmunoResearch Laboratories, West Grove, PA), the cells were permeabilized with 0.1% Triton X-100 for 10 minutes, blocked in 5% normal donkey serum for 1 hour and then incubated with anti-neurexin antibody (Santa Cruz Biotechnology, Santa Cruz, CA, 1:100) at 4°C overnight. Following three gentle washes, cells were incubated with fluorescein isothiocyanate-conjugated donkey anti-goat secondary antibody (Jackson ImmunoResearch Laboratories) for 1 hour. For immunocytochemical assays of the glutamate receptor subunit GluR2 (GluA2) and the presynaptic vesicular protein synapsin, cultured neurons at the ninth DIV were treated with AChE-conditioned medium or with AChE-free conditioned medium in the presence of 10 μM physostigmine. At the 12th DIV, the treated neurons were fixed with 3.7% paraformaldehyde and 4% sucrose in PBS for 15 minutes and were then permeabilized in 0.1% Triton X-100 for 10–15 minutes. Following 1 hour of blocking with 5% goat serum, the cells were incubated with anti-GluR2 or anti-synapsin (Chemicon, 1:1000).

### Confocal microscopy

Visual fields under a confocal microscope (Carl Zeiss, Gottingen, Germany) were randomly selected by blindly moving the cell-culture coverslip. Dual immunofluorescence was captured in two-channel mode, as previously described [76]. Digital images were obtained with a 63× objective lens. Multiple electronic images of cells were obtained and saved in a computer for analysis. For both control and treated neurons, the number of immunofluorescent protein particles was counted and the length of neurites was measured using Image J software (NIH Image, NIH, Bethesda, MD). This information was used to calculate the density of immunoreactive protein clusters, expressed as number of clusters per 20 μm length of dendrite. For colocalization analysis, confocal microscopic images of the two channels were background-subtracted and the threshold of fluorescent punctate structures at neuronal dendrites was set to the level at least twofold greater than the background. As previously described [77], colocalization was measured by means of Image J as the fraction of the image filed labeled for one channel that was also labeled for the second channel.

### Co-immunoprecipitation of neurexin and AChE from cultured neurons

At the seventh DIV, the cultured hippocampal neurons, with or without BW284c51 treatment (5 μM, for 3 days), were lysed in a buffer containing 20 mM Tris–HCl (pH 7.5), 150 mM NaCl, 0.5% Triton X-100 and protease inhibitors. Cell lysate (500–1000 μl, concentration 1 μg/μl) was incubated with Protein A-Sepharose beads (Sigma) at 4°C for 30 minutes to remove nonspecific binding proteins. The cleared lysate was then incubated with anti-neurexin antibody (Santa Cruz Biotechnology) or anti-AChE antibody (BD Biosciences, Franklin Lakes, NJ) overnight at 4°C, to allow reciprocal co-immunoprecipitation of neurexin and AChE. For controls, cell lysate was incubated with the same amount of normal IgG (Santa Cruz Biotechnology) or without added antibody. After incubation with Protein A-Sepharose beads for 2 hours, the immunoprecipitates were washed three times with ice-cold lysis buffers, and the binding proteins were eluted with sample buffer. The AChE inhibitor BW284c51 (5 μM) was included in all buffers used for immunoprecipitation. The general procedures for Western blotting were the same as previously described [21]. Briefly, after electro-transfer of the precipitates to nitrocellulose membrane (Bio-Rad, Hercules, CA), AChE or neurexin in the immunoprecipitated material was probed by immunoblotting with anti-AChE antibody (BD Biosciences) or anti-neurexin antibody (Santa Cruz Biotechnology).

### Transfection of HEK293 cells with neurexin-1β and hAChE cDNA

To study whether specific AChE isoforms interact with neurexin-1β, HEK293 cells were transiently transfected with plasmid DNA encoding neurexin-1β and specific isoforms of hAChE, using Lipofectamine 2000 transfection agent according to the manufacturer’s protocol. Briefly, HEK293 cells at 70%–80% confluence were co-transfected with cDNA encoding Nrxn-1β-1’-*His* and either hAChE-S or hAChE-R. As controls, some cells were transfected with Nrxn-1β-1’-*His*, hAChE-S or hAChE-S-R cDNA alone. To study whether AChE interacts extracellularly with Nrxn-1β-1’-*His* on the cell membrane, one set of HEK293 cells was transfected with hAChE-S and another set with Nrxn-1β-1’-*His*. Five hours later, the HEK293 cells transfected with Nrxn-1β-1’-*His* were gently stripped off and then re-seeded onto the HEK293 cells transfected with hAChE-S (or onto cells transfected with control vector), at a ratio of 1:1.5. For all transfection operations, equal amounts of transfected DNA were obtained by adding vector DNA, and the catalytic action of AChE was blocked by adding physostigmine (10 μM) to the medium.

### Characterization of transfected proteins in HEK293 cells

To detect AChE variants, HEK293 cells were lysed in a buffer containing 20 mM Tris–HCl (pH 7.5), 150 mM NaCl, 1% Triton X-100 and proteinase inhibitors. Immunoblotting of the cell lysate or the culture medium collected from the transfected cells was performed under non-reducing conditions. Specifically, the protein sample was applied to 10% gel using the loading buffer without the reducing agent, as described previously [78].

To study the glycosylation profiles of proteins expressed in HEK293 cells, protein samples obtained from the lysates of transfected cells or from the culture medium were denatured and incubated with peptide *N*-glycosidase F (PNGase F), *O*-glycosidase and /or sialidase using Enzymatic CarboRelease Kit (QA-Bio, Palm Desert, CA) for 3 hours at 37°C according to the manufacturer’s protocol and then processed for Western blotting. To study the effect of *N*-linked glycosylation on the interaction between AChE and β-neurexins, the transfected cells were treated (5 hours after transfection) with tunicamycin (0.5 μg/ml, Sigma), a selective inhibitor of *N*-glycosylase, or the equivalent volume of dimethyl sulfoxide (vehicle control). Cells were lysed 40–48 hours after transfection, and co-immunoprecipitation was performed as described above.

Membrane surface proteins in transfected HEK293 cells were examined by surface biotination of intact cells with EZ-Link Sulfo-NHS-SS-Biotin reagent (Pierce Biotechnology, Rockford, IL) according to the manufacturer’s protocol. Briefly, intact cells were washed and then incubated in the reagent (0.5 mg/ml in PBS, pH 8.0) for 1 hour on ice. Unbound biotin was quenched by incubation in 50 mM Tris, pH 8.0, for 10 minutes on ice. Cells were washed two times with ice-cold PBS and then lysed. The biotinylated proteins were precipitated by rocking with streptavidin beads (Pierce) overnight at 4°C. The beads were washed three times in lysis buffer, and bound proteins were eluted in sodium dodecyl sulfate sample buffer. The membrane proteins were confirmed by immunoblotting with specific antibodies.

### Co-immunoprecipitation of protein samples from transfected HEK293 cells

Transfected HEK293 cells were used for co-immunoprecipitation assays 40–48 hours after transfection. Briefly, the transfected cells were lysed in ice-cold 1% Triton X-100 and 0.5% sodium deoxycholate in PBS supplemented with protease inhibitors. AChE and neurexin were co-immunoprecipitated by incubating the cleared lysates with anti-AChE antibody (Santa Cruz Biotechnology) or anti-*His* antibody (QIAGEN, Hilden, Germany) for 3 hours at 4°C. Physostigmine (10 μM) was included in all buffers used for immunoprecipitation. Neurexin or AChE in the immunoprecipitated material was probed by immunoblotting with anti-*His* followed by anti-mouse light-chain-specific secondary antibody (Jackson ImmunoResearch Laboratories) or with AChE antibody (Santa Cruz Biotechnology). To control for efficiency of transfection, the expression levels of all proteins were assessed by Western blotting. The blot films were scanned with a GS800 densitometer (Bio-Rad).

To investigate whether increased extracellular AChE interrupts the neurexin–neuroligin association, HEK293 cells were co-transfected with cDNA encoding Nrxn-1β-1’-*His* and neuroligin-1, and the transfected HEK293 cells were cultured in AChE-S-conditioned medium or AChE-free conditioned medium. Twenty-four hours after co-transfection, HEK293 cells were lysed as previously described [17], with minor modification, in a buffer containing (in mM) 20 Tris–HCl (pH 7.5), 100 NaCl, 4 KCl and 5 CaCl_2_, as well as 2% CHAPS cell lysis buffer and protease inhibitors, at 4°C. The cell lysate was cleared as described above and then incubated with anti-*His* antibody (QIAGEN), and the precipitated neuroligin was confirmed by immunoblotting with anti-neuroligin antibody (Synaptic Systems, Göttingen, Germany).

### Co-culture of neurons with neuroligin-expressing HEK293 cells

HEK293 cells plated at low density on poly-D-lysine coated glass coverslips were cultured in DMEM. Five hours after plating, the medium was replaced with neuron plating medium, and dissociated E18 hippocampal cells (3 × 10 [4] cells/cm [2]) were plated onto the HEK293 cells. On the fifth day of co-culture, the cells were transfected with rat neuroligin-1 cDNA and the GFP-encoding vector, using Lipofectamine 2000 transfection agent. Five hours after transfection, the medium was replaced with AChE-conditioned medium or AChE-free conditioned medium in the presence of 10 μM physostigmine. Two days after transfection of neuroligin-1, the cells were processed according to the procedures used for neurons (see above) and then incubated with anti-synapsin (Chemicon, 1:1000) at 4°C overnight. Following incubation with Cy3-conjugated secondary antibodies at 4°C for 1 hour, the coverslips were rinsed and then mounted for confocal microscopic examination.

### Patch-clamp recordings in cultured neurons

Control and treated neurons were used for patch-clamp recordings 40–48 hours after treatment. Transfected neurons were identified by green fluorescence, and a sample of these neurons was randomly selected for recordings. The procedures for voltage-clamp recording were as previously described [21]. Briefly, recordings were obtained by means of an Axopatch-1D amplifier (Axon Instruments, Foster City, CA) with holding potential of −60 mV. The extracellular solution contained (in mM): 145 NaCl, 1.3 CaCl_2_, 5.4 KCl, 25 HEPES and 28 glucose, with pH 7.4 and osmolarity about 315 mOsm. The patch electrodes, made with thin-walled glass tubes, were filled with a solution containing (in mM) 150 KCl, 10 HEPES, 1 CaCl_2_, 2 MgCl_2_, 2 tetraethylammonium and 4 ATP, with pH 7.35 and osmolarity 310 mOsm. To study the effects of AChE on the function of the major transmitter receptors in neurons, glutamate-evoked and γ-aminobutyric acid (GABA)-evoked currents were studied. Rapid application of the receptor agonist was achieved with a computer-controlled, multibarrel-perfusion system (SF-77B, Warner Instruments, Hamden, CT). Electrical signals were digitized, filtered (1–2 kHz) and acquired on-line by means of the data acquisition and analysis software Clampex (Axon Instruments). The peak amplitude of evoked currents was measured off-line using Clampfit software (Axon Instruments). For recording of miniature excitatory postsynaptic currents (mEPSCs), 0.5 μM of tetrodotoxin and 20 μM of bicuculline methiodide were included in the ECS. All recordings were performed at room temperature (22-24°C). In each recording, at least 120- mEPSC events were collected for analysis. The amplitude and frequency of mEPSCs were analyzed using the program Mini-analysis (Synaptosoft Inc., Decatur, GA).

### Statistical analysis

Statistical analyses were performed using Student’s unpaired or paired *t* tests as appropriate. Data are expressed as mean ± standard error of the mean (SEM). A *p* value < 0.05 was considered significant.

## Competing interests

The authors declare that they have no competing interests.

## Authors’ contributions

YYX, HD, BBY and WYL performed experiments and conducted data analysis. BBY, JFM and WYL participated in experimental designs and results discussion. JFM and WYL drafted and edited the manuscripts. All authors read and approved the final manuscript.

## References

[B1] Soreq H Seidman S Acetylcholinesterase - new roles for an old actor Nat Rev Neurosci 2001 2 294 302 10.1038/35067589 11283752

[B2] Perrier AL Massoulie J Krejci E PRiMA: the membrane anchor of acetylcholinesterase in the brain Neuron 2002 33 275 285 10.1016/S0896-6273(01)00584-0 11804574

[B3] Hicks D John D Makova NZ Henderson Z Nalivaeva NN Turner AJ Membrane targeting, shedding and protein interactions of brain acetylcholinesterase J Neurochem 2011 116 742 746 10.1111/j.1471-4159.2010.07032.x 21214569

[B4] Johnston MV McKinney M Coyle JT Evidence for a cholinergic projection to neocortex from neurons in basal forebrain Proc Natl Acad Sci USA 1979 76 5392 5396 10.1073/pnas.76.10.5392 388436 PMC413149

[B5] Aubert I Poirier J Gauthier S Quirion R Multiple cholinergic markers are unexpectedly not altered in the rat dentate gyrus following entorhinal cortex lesions J Neurosci 1994 14 2476 2484 8182423 10.1523/JNEUROSCI.14-05-02476.1994 PMC6577437

[B6] Saez-Valero J Fodero LR White AR Barrow CJ Small DH Acetylcholinesterase is increased in mouse neuronal and astrocyte cultures after treatment with beta-amyloid peptides Brain Res 2003 965 283 286 10.1016/S0006-8993(02)04159-8 12591148

[B7] Coyle JT Price DL DeLong MR Alzheimer's disease: a disorder of cortical cholinergic innervation Science 1983 219 1184 1190 10.1126/science.6338589 6338589

[B8] Atack JR Perry EK Bonham JR Candy JM Perry RH Molecular forms of acetylcholinesterase and butyrylcholinesterase in the aged human central nervous system J Neurochem 1986 47 263 277 3711902 10.1111/j.1471-4159.1986.tb02858.x

[B9] Fishman EB Siek GC MacCallum RD Bird ED Volicer L Marquis JK Distribution of the molecular forms of acetylcholinesterase in human brain: alterations in dementia of the Alzheimer type Ann Neurol 1986 19 246 252 10.1002/ana.410190305 3963769

[B10] Navaratnam DS Priddle JD McDonald B Esiri MM Robinson JR Smith AD Anomalous molecular form of acetylcholinesterase in cerebrospinal fluid in histologically diagnosed Alzheimer's disease Lancet 1991 337 447 450 10.1016/0140-6736(91)93391-L 1671469

[B11] Talesa VN Acetylcholinesterase in Alzheimer's disease Mech Ageing Dev 2001 122 1961 1969 10.1016/S0047-6374(01)00309-8 11589914

[B12] Saez-Valero J Sberna G McLean CA Masters CL Small DH Glycosylation of acetylcholinesterase as diagnostic marker for Alzheimer's disease Lancet 1997 350 929 10.1016/S0140-6736(97)24039-0 9314873

[B13] Alvarez A Opazo C Alarcon R Garrido J Inestrosa NC Acetylcholinesterase promotes the aggregation of amyloid-beta-peptide fragments by forming a complex with the growing fibrils J Mol Biol 1997 272 348 361 10.1006/jmbi.1997.1245 9325095

[B14] Munoz FJ Inestrosa NC Neurotoxicity of acetylcholinesterase amyloid beta-peptide aggregates is dependent on the type of Abeta peptide and the AChE concentration present in the complexes FEBS Lett 1999 450 205 209 10.1016/S0014-5793(99)00468-8 10359075

[B15] Rees T Hammond PI Soreq H Younkin S Brimijoin S Acetylcholinesterase promotes beta-amyloid plaques in cerebral cortex Neurobiol Aging 2003 24 777 787 10.1016/S0197-4580(02)00230-0 12927760

[B16] Selkoe DJ Alzheimer’s disease is a synaptic failure Science 2002 298 789 791 10.1126/science.1074069 12399581

[B17] Ichtchenko K Hata Y Nguyen T Ullrich B Missler M Moomaw C Sudhof TC Neuroligin 1: a splice site-specific ligand for beta-neurexins Cell 1995 81 435 443 10.1016/0092-8674(95)90396-8 7736595

[B18] Song JY Ichtchenko K Sudhof TC Brose N Neuroligin 1 is a postsynaptic cell-adhesion molecule of excitatory synapses Proc Natl Acad Sci USA 1999 96 1100 1105 10.1073/pnas.96.3.1100 9927700 PMC15357

[B19] Dean C Dresbach T Neuroligins and neurexins: linking cell adhesion, synapse formation and cognitive function Trends Neurosci 2006 29 21 29 16337696 10.1016/j.tins.2005.11.003

[B20] Scholl FG Scheiffele P Making connections: cholinesterase-domain proteins in the CNS Trends Neurosci 2003 26 618 624 10.1016/j.tins.2003.09.004 14585602

[B21] Dong H Xiang YY Farchi N Ju W Wu Y Chen L Wang Y Hochner B Yang B Soreq H Lu WY Excessive expression of acetylcholinesterase impairs glutamatergic synaptogenesis in hippocampal neurons Trends Neurosci 2004 24 8950 8960 10.1523/JNEUROSCI.2106-04.2004 PMC6730061 15483114

[B22] Rotundo RL Carbonetto ST Neurons segregate clusters of membrane-bound acetylcholinesterase along their neurites Proc Natl Acad Sci USA 1987 84 2063 2067 10.1073/pnas.84.7.2063 3470777 PMC304585

[B23] Dean C Scholl FG Choih J DeMaria S Berger J Isacoff E Scheiffele P Neurexin mediates the assembly of presynaptic terminals Nat Neurosci 2003 6 708 716 10.1038/nn1074 12796785 PMC1646425

[B24] Friedman A Kaufer D Shemer J Hendler I Soreq H Tur-Kaspa I Pyridostigmine brain penetration under stress enhances neuronal excitability and induces early immediate transcriptional response Nat Med 1996 2 1382 1385 10.1038/nm1296-1382 8946841

[B25] Meshorer E Soreq H Virtues and woes of AChE alternative splicing in stress-related neuropathologies Trends Neurosci 2006 29 216 224 10.1016/j.tins.2006.02.005 16516310

[B26] Chitlaru T Kronman C Zeevi M Kam M Harel A Ordentlich A Velan B Shafferman A Modulation of circulatory residence of recombinant acetylcholinesterase through biochemical or genetic manipulation of sialylation levels Biochem J 1998 336 Pt 3 647 658 9841877 10.1042/bj3360647 PMC1219916

[B27] Velan B Grosfeld H Kronman C Leitner M Gozes Y Lazar A Flashner Y Marcus D Cohen S Shafferman A The effect of elimination of intersubunit disulfide bonds on the activity, assembly, and secretion of recombinant human acetylcholinesterase. Expression of acetylcholinesterase Cys-580––Ala mutant J Biol Chem 1991 266 23977 23984 1748670

[B28] Randall WR Cellular expression of a cloned, hydrophilic, murine acetylcholinesterase. Evidence of palmitoylated membrane-bound forms J Biol Chem 1994 269 12367 12374 7512968

[B29] Mendelson I Kronman C Ariel N Shafferman A Velan B Bovine acetylcholinesterase: cloning, expression and characterization Biochem J 1998 334 Pt 1 251 259 9693127 10.1042/bj3340251 PMC1219686

[B30] Chitlaru T Kronman C Velan B Shafferman A Overloading and removal of N-glycosylation targets on human acetylcholinesterase: effects on glycan composition and circulatory residence time Biochem J 2002 363 619 631 10.1042/0264-6021:3630619 11964163 PMC1222515

[B31] Velan B Kronman C Ordentlich A Flashner Y Leitner M Cohen S Shafferman A N-glycosylation of human acetylcholinesterase: effects on activity, stability and biosynthesis Biochem J 1993 296 Pt 3 649 656 8280063 10.1042/bj2960649 PMC1137746

[B32] Andres C Beeri R Friedman A Lev-Lehman E Henis S Timberg R Shani M Soreq H Acetylcholinesterase-transgenic mice display embryonic modulations in spinal cord choline acetyltransferase and neurexin Ibeta gene expression followed by late-onset neuromotor deterioration Proc Natl Acad Sci U S A 1997 94 8173 8178 10.1073/pnas.94.15.8173 9223334 PMC21576

[B33] Ushkaryov YA Hata Y Ichtchenko K Moomaw C Afendis S Slaughter CA Sudhof TC Conserved domain structure of beta-neurexins. Unusual cleaved signal sequences in receptor-like neuronal cell-surface proteins J Biol Chem 1994 269 11987 11992 8163501

[B34] Graf ER Kang Y Hauner AM Craig AM Structure function and splice site analysis of the synaptogenic activity of the neurexin-1 beta LNS domain J Neurosci 2006 26 4256 4265 10.1523/JNEUROSCI.1253-05.2006 16624946 PMC2826202

[B35] Shafferman A Ordentlich A Barak D Kronman C Ber R Bino T Ariel N Osman R Velan B Electrostatic attraction by surface charge does not contribute to the catalytic efficiency of acetylcholinesterase EMBO J 1994 13 3448 3455 8062821 10.1002/j.1460-2075.1994.tb06650.x PMC395247

[B36] Radic Z Kirchhoff PD Quinn DM McCammon JA Taylor P Electrostatic influence on the kinetics of ligand binding to acetylcholinesterase. Distinctions between active center ligands and fasciculin J Biol Chem 1997 272 23265 23277 10.1074/jbc.272.37.23265 9287336

[B37] Paraoanu LE Layer PG Mouse acetylcholinesterase interacts in yeast with the extracellular matrix component laminin-1beta FEBS Lett 2004 576 161 164 10.1016/j.febslet.2004.08.078 15474030

[B38] Sperling LE Klaczinski J Schutz C Rudolph L Layer PG Mouse acetylcholinesterase enhances neurite outgrowth of rat R28 cells through interaction with laminin-1 PLoS One 2012 7 e36683 10.1371/journal.pone.0036683 22570738 PMC3343015

[B39] Scheiffele P Fan J Choih J Fetter R Serafini T Neuroligin expressed in nonneuronal cells triggers presynaptic development in contacting axons Cell 2000 101 657 669 10.1016/S0092-8674(00)80877-6 10892652

[B40] Silman I Sussman JL Acetylcholinesterase: ‘classical’ and ‘non-classical’ functions and pharmacology Curr Opin Pharmacol 2005 5 293 302 10.1016/j.coph.2005.01.014 15907917

[B41] Layer PG Weikert T Alber R Cholinesterases regulate neurite growth of chick nerve cells in vitro by means of a non-enzymatic mechanism Cell Tissue Res 1993 273 219 226 10.1007/BF00312823 8103422

[B42] Bytyqi AH Lockridge O Duysen E Wang Y Wolfrum U Layer PG Impaired formation of the inner retina in an AChE knockout mouse results in degeneration of all photoreceptors Eur J Neurosci 2004 20 2953 2962 10.1111/j.1460-9568.2004.03753.x 15579149

[B43] Beeri R Le Novere N Mervis R Huberman T Grauer E Changeux JP Soreq H Enhanced hemicholinium binding and attenuated dendrite branching in cognitively impaired acetylcholinesterase-transgenic mice J Neurochem 1997 69 2441 2451 9375677 10.1046/j.1471-4159.1997.69062441.x

[B44] Andres C Seidman S Beeri R Timberg R Soreq H Transgenic acetylcholinesterase induces enlargement of murine neuromuscular junctions but leaves spinal cord synapses intact Neurochem Int 1998 32 449 456 10.1016/S0197-0186(97)00121-6 9676744

[B45] Layer PG Weikert T Willbold E Chicken retinospheroids as developmental and pharmacological in vitro models: acetylcholinesterase is regulated by its own and by butyrylcholinesterase activity Cell Tissue Res 1992 268 409 418 10.1007/BF00319147 1628298

[B46] Comoletti D Flynn R Jennings LL Chubykin A Matsumura T Hasegawa H Sudhof TC Taylor P Characterization of the interaction of a recombinant soluble neuroligin-1 with neurexin-1beta J Biol Chem 2003 278 50497 50505 10.1074/jbc.M306803200 14522992

[B47] Inestrosa NC Alvarez A Perez CA Moreno RD Vicente M Linker C Casanueva OI Soto C Garrido J Acetylcholinesterase accelerates assembly of amyloid-beta-peptides into Alzheimer's fibrils: possible role of the peripheral site of the enzyme Neuron 1996 16 881 891 10.1016/S0896-6273(00)80108-7 8608006

[B48] Berson A Knobloch M Hanan M Diamant S Sharoni M Schuppli D Geyer BC Ravid R Mor TS Nitsch RM Soreq H Changes in read through acetylcholinesterase expression modulate amyloid-beta pathology Brain 2008 131 109 119 18056160 10.1093/brain/awm276

[B49] Sussman JL Harel M Frolow F Oefner C Goldman A Toker L Silman I Atomic structure of acetylcholinesterase from Torpedo californica: a prototypic acetylcholine-binding protein Science 1991 253 872 879 10.1126/science.1678899 1678899

[B50] Johnson G Moore SW Human acetylcholinesterase binds to mouse laminin-1 and human collagen IV by an electrostatic mechanism at the peripheral anionic site Neurosci Lett 2003 337 37 40 10.1016/S0304-3940(02)01298-3 12524166

[B51] Barak D Kronman C Ordentlich A Ariel N Bromberg A Marcus D Lazar A Velan B Shafferman A Acetylcholinesterase peripheral anionic site degeneracy conferred by amino acid arrays sharing a common core J Biol Chem 1994 269 6296 6305 8119978

[B52] Arac D Boucard AA Ozkan E Strop P Newell E Sudhof TC Brunger AT Structures of neuroligin-1 and the neuroligin-1/neurexin-1 beta complex reveal specific protein-protein and protein-Ca2+ interactions Neuron 2007 56 992 1003 10.1016/j.neuron.2007.12.002 18093522

[B53] Krall WJ Sramek JJ Cutler NR Cholinesterase inhibitors: a therapeutic strategy for Alzheimer disease Ann Pharmacother 1999 33 441 450 10.1345/aph.18211 10332536

[B54] Layer PG Cholinesterases preceding major tracts in vertebrate neurogenesis Bioessays 1990 12 415 420 10.1002/bies.950120904 2256905

[B55] Geula C Mesulam MM Kuo CC Tokuno H Postnatal development of cortical acetylcholinesterase-rich neurons in the rat brain: permanent and transient patterns Exp Neurol 1995 134 157 178 10.1006/exnr.1995.1046 7556536

[B56] Forloni G Blake K Hohmann CH Coyle JT The postnatal expression of acetylcholinesterase in somatostatin-positive cells of mouse hippocampus Brain Res Dev Brain Res 1989 48 73 85 10.1016/0165-3806(89)90094-1 2752576

[B57] Tagliari B Tagliari AP Schmitz F da Cunha AA Dalmaz C Wyse AT Chronic variable stress alters inflammatory and cholinergic parameters in hippocampus of rats Neurochem Res 2011 36 487 493 10.1007/s11064-010-0367-0 21184279

[B58] Mavanji V Datta S Clomipramine treatment in neonatal rats alters the brain acetylcholinesterase activity in adulthood Neurosci Lett 2002 330 119 121 10.1016/S0304-3940(02)00725-5 12213647

[B59] Adamec R Head D Soreq H Blundell J The role of the read through variant of acetylcholinesterase in anxiogenic effects of predator stress in mice Behav Brain Res 2008 189 180 190 10.1016/j.bbr.2007.12.023 18243359

[B60] Beeri R Andres C Lev-Lehman E Timberg R Huberman T Shani M Soreq H Transgenic expression of human acetylcholinesterase induces progressive cognitive deterioration in mice Curr Biol 1995 5 1063 1071 10.1016/S0960-9822(95)00211-9 8542283

[B61] Chih B Engelman H Scheiffele P Control of excitatory and inhibitory synapse formation by neuroligins Science 2005 307 1324 1328 10.1126/science.1107470 15681343

[B62] Graf ER Zhang X Jin SX Linhoff MW Craig AM Neurexins induce differentiation of GABA and glutamate postsynaptic specializations via neuroligins Cell 2004 119 1013 1026 10.1016/j.cell.2004.11.035 15620359 PMC2826211

[B63] Scheiffele P Cell-cell signaling during synapse formation in the CNS Ann Rev Neurosci 2003 26 485 508 10.1146/annurev.neuro.26.043002.094940 12626697

[B64] Sudhof TC Neuroligins and neurexins link synaptic function to cognitive disease Nature 2008 455 903 911 10.1038/nature07456 18923512 PMC2673233

[B65] Shaked I Meerson A Wolf Y Avni R Greenberg D Gilboa-Geffen A Soreq H MicroRNA-132 potentiates cholinergic anti-inflammatory signaling by targeting acetylcholinesterase Immunity 2009 31 965 973 10.1016/j.immuni.2009.09.019 20005135

[B66] Lau P Bossers K Janky R Salta E Frigerio CS Barbash S Rothman R Sierksma AS Thathiah A Greenberg D Papadopoulou AS Achsel T Ayoubi T Soreq H Verhaagen J Swaab DF Aerts S De SB Alteration of the microRNA network during the progression of Alzheimer's disease EMBO Mol Med 2013 5 1613 1634 10.1002/emmm.201201974 24014289 PMC3799583

[B67] Perry EK Lee ML Martin-Ruiz CM Court JA Volsen SG Merrit J Folly E Iversen PE Bauman ML Perry RH Wenk GL Cholinergic activity in autism: abnormalities in the cerebral cortex and basal forebrain Am J Psychiatry 2001 158 1058 1066 10.1176/appi.ajp.158.7.1058 11431227

[B68] Sternfeld M Ming G Song H Sela K Timberg R Poo M Soreq H Acetylcholinesterase enhances neurite growth and synapse development through alternative contributions of its hydrolytic capacity, core protein, and variable C termini J Neurosci 1998 18 1240 1249 9454834 10.1523/JNEUROSCI.18-04-01240.1998 PMC6792736

[B69] Ichtchenko K Nguyen T Sudhof TC Structures, alternative splicing, and neurexin binding of multiple neuroligins J Biol Chem 1996 271 2676 2682 10.1074/jbc.271.5.2676 8576240

[B70] Sugita S Khvochtev M Sudhof TC Neurexins are functional alpha-latrotoxin receptors Neuron 1999 22 489 496 10.1016/S0896-6273(00)80704-7 10197529

[B71] Yang BL Cao L Kiani C Lee V Zhang Y Adams ME Yang BB Tandem repeats are involved in G1 domain inhibition of versican expression and secretion and the G3 domain enhances glycosaminoglycan modification and product secretion via the complement-binding protein-like motif J Biol Chem 2000 275 21255 21261 10.1074/jbc.M001443200 10801813

[B72] Rodriguez MS Desterro JM Lain S Midgley CA Lane DP Hay RT SUMO-1 modification activates the transcriptional response of p53 EMBO J 1999 18 6455 6461 10.1093/emboj/18.22.6455 10562557 PMC1171708

[B73] Velan B Kronman C Grosfeld H Leitner M Gozes Y Flashner Y Sery T Cohen S Ben Aziz R Seidman S Recombinant human acetylcholinesterase is secreted from transiently transfected 293 cells as a soluble globular enzyme Cell Mol Neurobiol 1991 11 143 156 10.1007/BF00712806 1849451 PMC11567368

[B74] Bae JJ Xiang YY Martinez-Canabal A Frankland PW Yang BB Lu WY Increased transforming growth factor-beta1 modulates glutamate receptor expression in the hippocampus Int J Physiol Pathophysiol Pharmacol 2011 3 9 20 21479098 PMC3068849

[B75] Brewer GJ Torricelli JR Evege EK Price PJ Optimized survival of hippocampal neurons in B27-supplemented Neurobasal, a new serum-free medium combination J Neurosci Res 1993 35 567 576 10.1002/jnr.490350513 8377226

[B76] Xiang YY Dong H Wan Y Li J Yee A Yang BB Lu WY Versican G3 domain regulates neurite growth and synaptic transmission of hippocampal neurons by activation of epidermal growth factor receptor J Biol Chem 2006 281 19358 19368 10.1074/jbc.M512980200 16648628

[B77] Flores CE Cachope R Nannapaneni S Ene S Nairn AC Pereda AE Variability of distribution of Ca(2+)/calmodulin-dependent kinase II at mixed synapses on the mauthner cell: colocalization and association with connexin 35 J Neurosci 2010 30 9488 9499 10.1523/JNEUROSCI.4466-09.2010 20631177 PMC2945303

[B78] Darreh-Shori T Hellstrom-Lindahl E Flores-Flores C Guan ZZ Soreq H Nordberg A Long-lasting acetylcholinesterase splice variations in anticholinesterase-treated Alzheimer's disease patients J Neurochem 2004 88 1102 1113 10.1046/j.1471-4159.2003.02230.x 15009666

